# Anion binding to a cationic europium(iii) probe enables the first real-time assay of heparan sulfotransferase activity†

**DOI:** 10.1039/d1ob02071d

**Published:** 2021-12-08

**Authors:** Simon Wheeler, Colum Breen, Yong Li, Sarah H. Hewitt, Erin Robertson, Edwin A. Yates, Igor L. Barsukov, David G. Fernig, Stephen J. Butler

**Affiliations:** Department of Chemistry, Loughborough University Epinal Way Loughborough LE11 3TU UK s.j.butler@lboro.ac.uk; Department of Biochemistry, Institute for Integrative Biology, University of Liverpool Liverpool L69 7ZB UK

## Abstract

Sulfotransferases constitute a ubiquitous class of enzymes which are poorly understood due to the lack of a convenient tool for screening their activity. These enzymes use the anion PAPS (adenosine-3′-phosphate-5′-phosphosulfate) as a donor for a broad range of acceptor substrates, including carbohydrates, producing sulfated compounds and PAP (adenosine-3′,5′-diphosphate) as a side product. We present a europium(iii)-based probe that binds reversibly to both PAPS and PAP, producing a larger luminescence enhancement with the latter anion. We exploit this greater emission enhancement with PAP to demonstrate the first direct real-time assay of a heparan sulfate sulfotransferase using a multi-well plate format. The selective response of our probe towards PAP over structurally similar nucleoside phosphate anions, and over other anions, is investigated and discussed. This work opens the possibility of investigating more fully the roles played by this enzyme class in health and disease, including operationally simple inhibitor screening.

## Abstract 2

We present a new luminescent europium(iii)-based anion receptor that binds to PAP (adenosine-3′,5′-diphosphate). The increased emission intensity and lifetime of the receptor-PAP complex was used to develop the first real-time assay of heparan sulfotransferase activity.

## Graphical abstract

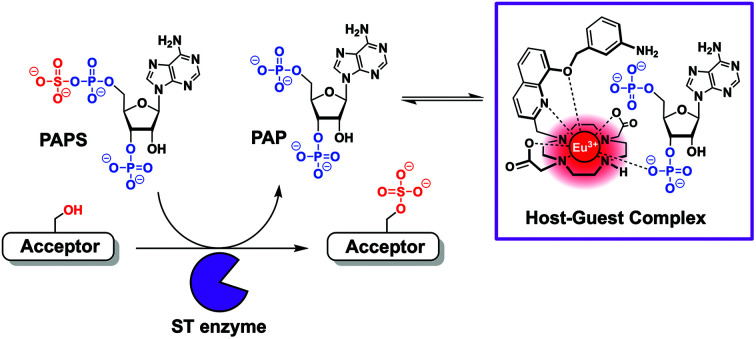

## Introduction

The past two decades have seen a multitude of studies aimed at fundamental understanding of lanthanide complexes and at their exploitation as luminescent sensors and probes.^1–7^ When suitably ligated and irradiated with light in the UV or visible range, certain lanthanide(iii) ions undergo an electronic excitation; relaxation of this excited state leads to luminescence.^8^ Such lanthanide complexes possess a range of qualities that make them particularly interesting and useful for sensing and imaging purposes, including their typically large pseudo-Stokes shifts, narrow emission linewidths and long emission lifetimes (millisecond range for europium and terbium).^9–12^ Anion responsive lanthanide complexes may be designed by introducing one or more vacant coordination sites at the metal centre; these are occupied by water molecules in aqueous solution which deactivate the Ln(iii) excited state through vibration of their O–H bonds. Thus, coordination of a target anion at the metal centre displaces one or more water molecules, leading to an extension of the excited state lifetime and increase of the luminescence. Careful design of the ligating group(s) can give rise to remarkable selectivity amongst anions, allowing sensors to be created that signal anion binding through modulation of emission intensity, lifetime and spectral form.^1,6^ In particular, it has proved possible to discriminate between very similar phosphate-containing biomolecules in complex aqueous solutions, an area where we have been active.^13–17^

**Scheme 1:**
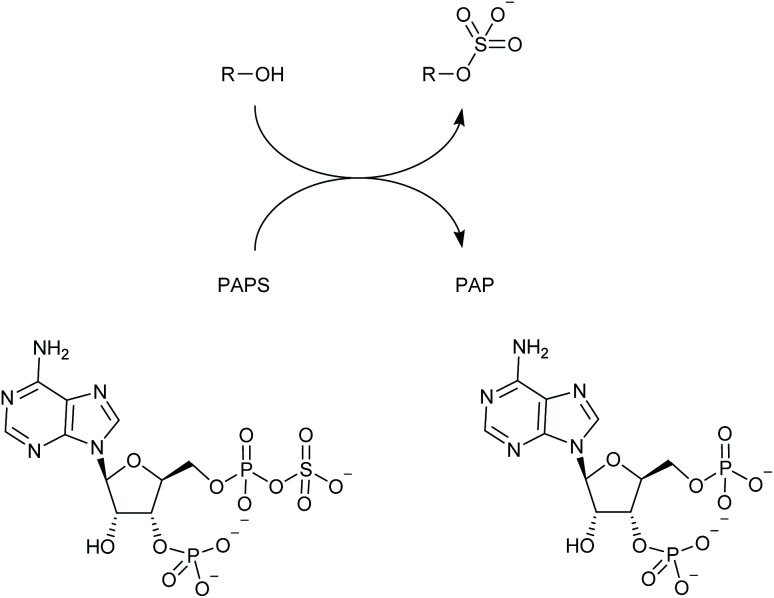
Sulfotransferases add SO_3_^−^ to hydroxyl or amino groups producing organic sulfates; PAPS functions as the donor yielding PAP as the by-product.

Heparan sulfotransferases mediate the attachment of a sulfate group to an atom (usually oxygen, though sometimes nitrogen) in the heparanosan/heparan sulfate polysaccharide (Scheme 1). They exist in numerous isoforms with varying substrate preferences.^18^ The sulfated products of this reaction play important roles in cell communication^19^ and also feature in various pathologies including those of cancer,^20^ Alzheimer's^21^ and the mucopolysaccharidoses.^22^ Heparan sulfotransferases belong to the wider group of sulfotransferases found across nature that use the universal sulfate donor compound PAPS (adenosine-3′-phosphate-5′-phosphosulfate),^23^ and produce PAP (adenosine-3′,5′-diphosphate) as a by-product (Scheme 1).

Very few methods have been disclosed for monitoring directly the activity of carbohydrate sulfotransferases. Those that have require radio-labelled substrates^24–26^ or specialized, expensive equipment and acceptor substrates.^27^ The majority of published assays examine endpoints making the accurate determination of enzyme kinetics difficult and time consuming. Very few are adaptable to high throughput format, accounting for the small numbers of reports of inhibitors of these enzymes.^24,28^ Notably, a recent study by Eyers and co-workers demonstrated that sulfotransferases inhibitors can be identified in specialised high-throughput screens.^27^ This work highlights that such high-throughput acceptor-based ST assays are limited in the acceptors that can be used, so the range of sulfotransferases that can be studied is restricted. Further investigation of heparan sulfotransferases, including the vast potential of inhibitors as targets for drug discovery across a range of therapeutic areas, will likely remain slow unless an assay capable of both kinetic measurements and automated high throughput screening can be developed. We postulated that an assay based on emissive europium(iii) probes could successfully fill this gap we had identified.

We recently developed a new class of cationic europium(iii) complexes bearing a sterically demanding 8-(benzyloxy)quinoline pendant arm that chelates the Eu(iii) ion, offering a single coordination site at the metal centre suitable for the monodentate binding of inorganic phosphate and AMP (adenosine monophosphate).^29^ We hypothesised that complexes of this design may be suitable for binding the structurally related phosphoanion, PAP, whilst showing a lower affinity for PAPS owing to the presence of the terminal cosulfate which would interact weakly with the hard Lewis acidic Eu(iii) centre. Thus, we envisaged that PAP would induce a larger emission enhancement compared with PAPS or with any sulfated products, allowing us to monitor the PAP/PAPS ratio during the progress of the sulfotransferase reaction. Our approach to monitoring sulfotransferase activity differs from previous work in that we target the small phosphoanion co-factor and the by-product derived from it, whereas other efforts have targeted the sulfated macromolecules or use an indirect coupled assay. Supramolecular approaches utilising cucurbituril and calixarene host molecules have been developed for monitoring enzyme reactions, such as hydrolases and methyltransferases^30–32^ while we have previously used supramolecular anion recognition of phosphate-based species to monitor kinases.^13,14^ Here we report the initial results of our investigations into heparan sulfotransferases and disclose a europium(iii)-based probe capable of monitoring one such enzyme in real-time, in principle independent of both substrate and product and so applicable to any sulfotransferase catalysed reaction.

## Results and discussion

**Fig. 1:**
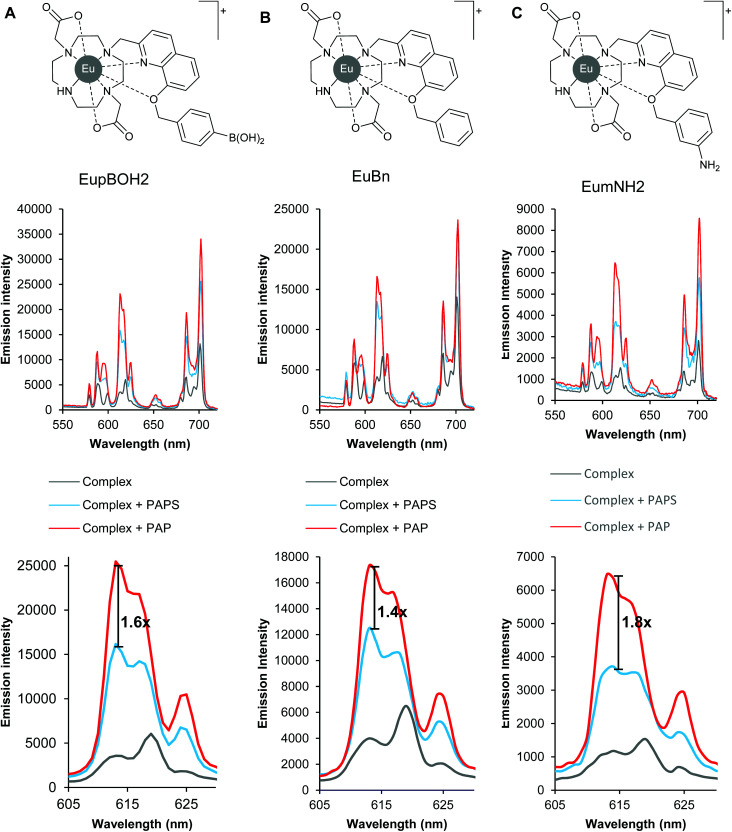
Structures of cationic Eu(iii) complexes and their emission responses alone and in the presence of PAPS and PAP. All three Eu(iii) probes give greater emission enhancements upon binding PAP compared with PAPS (A–C); removal of the boronate group resulted in a loss of discrimination (B *vs.* A) while installing an amino group increased it (C *vs.* A, B). All experiments conducted using 5 μM Eu(iii) complex, 250 μM anion in 50 mM TRIS at pH 7.4, rt.

We began our work by examining the binding of PAP and PAPS to two monocationic lanthanide complexes (Fig. 1) previously developed by us.^29^ These have been shown to bind relatively strongly to inorganic phosphate and AMP in aqueous solution, but do not bind sulfate, ATP, bicarbonate, lactate or acetate. Thus these complexes represented good starting points for the development of a luminescent probe for recognition of the structurally similar phosphoanion, PAP. We postulated that substituents on the distal benzene ring might affect the emission enhancements we observed in the presence of PAP and PAPS and thus complex EuBn, lacking the boronate ester group of EupBOH2, serves as an appropriate control complex to test our hypothesis.

Initially, we determined whether these two complexes could distinguish between PAP and PAPS by a differential luminescence response in 50 mM TRIS buffer at pH 7.4. Under these conditions, both anions induced an increase in overall emission intensity of the two complexes, with the largest emission change occurring in the Δ*J* = 2 (605–630 nm) emission band. Gratifyingly, PAP enhanced the emission of both complexes more than PAPS, as we expected. Compound EupBOH2 offered higher discrimination (Fig. 1A) between PAP and PAPS, giving a 1.6-fold difference in emission intensity at 613 nm. For justification of our use of this wavelength rather than other measures of the level of discrimination see Fig. S1.† In comparison, the binding of PAPS and PAP to complex EuBn gave a slightly reduced discrimination of 1.4-fold at 613 nm (Fig. 1B).

**Scheme 2:**
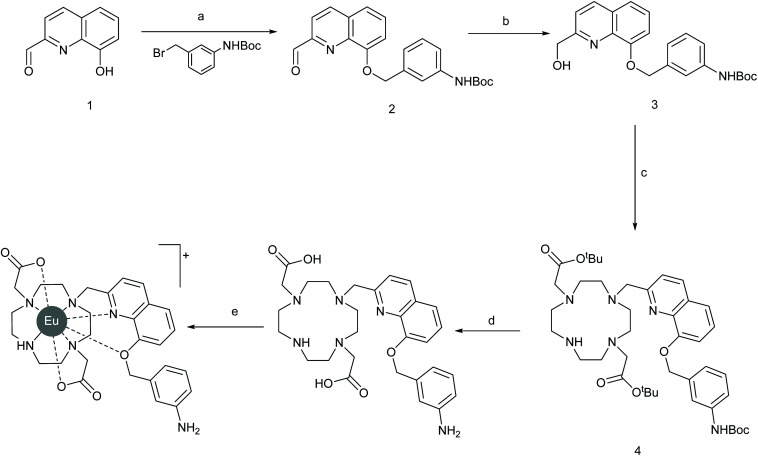
Synthesis of EumNH2. Conditions: (a) K_2_CO_3_, DMF, rt, 2 h, 88%; (b) NaBH_4_ MeOH, 0 °C to rt, 55%; (c) i) MsCl, Et_3_N, DCM, 0 °C to rt, (ii) DO2A *tert*-butyl ester, K_2_CO_3_, MeCN, 60 °C, 18hr, 71% over two steps; (d) 3 : 1 DCM/TFA, 18 h, quant; (e) EuCl_3_·6H_2_O, water, 60 °C, pH ≥ 7.2, prep HPLC, 9%.

Binding constants were determined for these complexes with PAP and PAPS in aqueous buffer at pH 7.4, by plotting the change in the intensity ratio of the Δ*J* = 2/Δ*J* = 1 bands against anion concentration and fitting the data to a 1 : 1 binding model. The binding titration data (Fig. S2†) indicated that there are only small differences in binding affinity of PAPS and PAP for either complex. Nevertheless, we considered our idea that substituents on the benzene ring affect anion discrimination to be vindicated and thus we set out to prepare a new complex EumNH2 functionalised with a *meta*-amino group on the benzene ring. Synthesis (Scheme 2) started with the alkylation of known^33^ phenol 1 with the requisite commercially available bromide followed by reduction of the resulting aldehyde 2 reduction to yield alcohol 3. Activation of this gave the slightly unstable mesylate ester which was thus used immediately in an alkylation reaction with known^34^ macrocycle to give protected ligand 4. Simultaneous deprotection of the *tert*-butyl esters and Boc group with TFA yielded the ligand 5 which was heated with EuCl_3_ to give our desired complex after purification by reverse-phase HPLC.

Photophysical characterisation of EumNH2 (Table 1, Fig. S3†) revealed a broad absorption band centred at 322 nm, identical to the unsubstituted complex EuBn and with a very similar extinction coefficient. The emission spectrum was also similar to EuBn, characterised by two components in the Δ*J* = 1 (585–600 nm) emission band and three components in the Δ*J* = 2 (605–630 nm) band. We suspected that the much lower quantum yield of EumNH2 and its smaller emission lifetime in water and D_2_O (Table 1, Table S1†) was caused by intramolecular photoinduced electron transfer (PET) from the nitrogen lone pair on the aniline ring quenching the europium excited state.

**Table 1:** Photophysical parameters of the Eu(iii) probes measured in water at 295 K For full data see Table S1†

	EupBOH2	EuBn	EumNH2
*λ*_max_ (nm)	322	322	322
*ε* (M^−1^ cm^−1^)	2900	2900	2500
*Φ* a (%)	1.2	1.5	0.3
*τ*_H_2_O _ b (ms)	0.18	0.17	0.05
*τ*_D_2_O _ b (ms)	0.25	0.23	0.07
*q* c	1.29	0.76	—d

a Quantum yields were calculated relative to quinine sulfate standard (*Φ*_em_ = 0.58); errors in quantum yields are ±20%.

b Lifetime experiments conducted using 50 μM probe, *λ*_ex_ = 322 nm, *λ*_em_ = 620 nm.

c Hydration state, *q*, was estimated using literature methods (errors are ±20%).

d Very short emission lifetimes prevented accurate estimation of the hydration state.

Consistently, the emission from this complex was found to be highly pH-sensitive with much higher emission intensity observed at lower pH values (Fig. S6†). Further support for the involvement of the aniline nitrogen lone pair was the presence of an absorption band centred at 292 nm which is absent from the spectrum of the parent complex (compare Fig. S3 and S4†) and which disappears at low pH (Fig. S6†). We assign this band to an n → π* transition. We attribute the emission enhancement and spectral form changes observed above pH 8 (Fig. S6†) to the coordination of hydroxide, but importantly the emission spectrum is stable in the pH range 6–8, rendering this compound potentially suitable for sulfotransferase assays.

The emission lifetime of the parent and boronic acid complexes in water were similar and extended in D_2_O as expected (Table 1 and S1†), giving rise to an estimation^35^ of the number of bound water molecules, *q*, of 1 within experimental error. We interpret these non-integer values as suggesting dynamic behaviour of the bound waters, including interaction with outer sphere molecules. The emission lifetime of complex EumNH2 was much shorter, consistent with PET quenching by the aniline nitrogen lone pair. Estimation of the *q* value for such complexes becomes difficult as the emission lifetime begins to overlap with the water exchange timescale.^35^ It is nonetheless significant that the emission lifetime was longer in D_2_O suggesting that the complex is hydrated in the absence of anions. When the overall emission intensity was measured in D_2_O it was found to be 2.9 times higher than in H_2_O, consistent with energy transfer to O–H vibrations being a significant non-radiative quenching pathway (Fig. S6†).

We were pleased to discover that, despite being less emissive, the aniline complex EumNH2 outperformed both our initial boronic acid and unsubstituted benzene complex showing a 1.8-fold discrimination in emission between PAP and PAPS at 613 nm (Fig. 1C). Analysis of the anion binding titration data (Fig. S1†) indicated that EumNH2 has a genuine preference for binding PAP over PAPS (log *K*_a_ = 4.0 and 3.5, respectively), albeit with a modest 5-fold selectivity for the former anion.

We wished to confirm our hypothesis that the phosphate groups in PAPS and PAP coordinate to the Eu(iii) ion displacing the bound water, and are thus responsible for the observed enhancement in emission and change in spectral form. First, we measured the emission response of EumNH2 in the presence of a small range of anions and found that inorganic phosphate was unique in giving a significant increase in luminescence especially in the Δ*J* = 2 band; other anions (sulfate, bicarbonate, nitrate, lactate and chloride) gave negligible changes in luminescence (Fig. S7†), indicating that these anions do not bind to EumNH2. Such high selectivity for phosphate was observed recently for structurally related Eu(iii) receptors containing a bulky 8-(benzyloxy)quinoline pendant arm that chelates the Eu(iii) ion, leaving a single coordination site for the phosphate binding.^29^

Other oxyanions that prefer a bidentate binding mode, including bicarbonate and lactate,^36^ do not bind to the receptors due to the steric hindrance imposed by the ligand, which blocks the ‘axial’ coordination site. The emission spectral form changes observed for EumNH2 in the presence of inorganic phosphate (Fig. S7†) matched that of PAP and PAPS, confirming that the phosphate-Eu(iii) coordination is the primary interaction involved in the host–guest complexes involving PAP or PAPS.

**Fig. 2:**
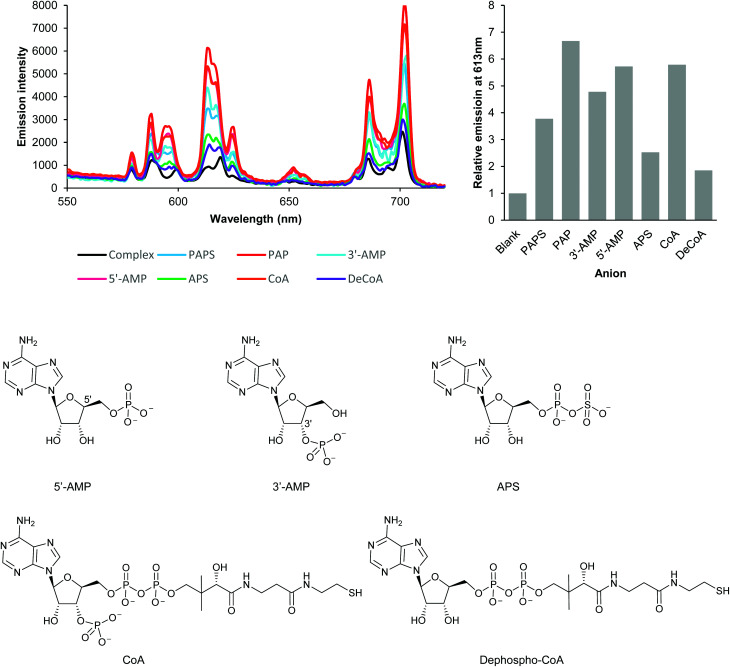
Investigation of anion binding modes by analysis of changes in the emission intensity and spectral form of EumNH2. Emission enhancement occurs if the anion possesses either a 3′-phosphate (PAPS, 3′-AMP, CoA), a 5′-phosphate (5′-CoA) or both (PAP); the absence of both 3′- and 5′-phosphates (APS, dephospho-CoA) results in very limited emission enhancement. Experiments conducted using 5 μM Eu(iii) complex, 250 μM anion in 50 mM TRIS at pH 7.4, 295 K. Very similar results were obtained for EupBOH2 and EuOBn (see Fig. S8†).

It is possible that addition of PAP and PAPS also leads to suppression of PeT quenching, contributing to the overall enhancement in emission intensity.^37,38^ If blocking of PET, rather than water displacement, was the main mechanism for the emission response then we would expect similar emission enhancements on binding anions in H_2_O and D_2_O. In fact, we observed a 2.8-fold increase in overall intensity on binding PAP in H_2_O but only 1.3-fold in D_2_O; binding PAPS gave a 2.0-fold increase in H_2_O but only 1.2-fold in D_2_O (Fig. S6†). The smaller enhancements in emission in D_2_O can be attributed to reduced quenching effect of O–D *versus* O–H oscillators. Additionally, suppression of PET by lowering the pH did not lead to changes in spectral form (Fig. S6†) unlike binding anions (Fig. 1, 2 and S7†). We thus conclude that the emission enhancements seen with PAP and PAPS are predominantly due to displacement of bound water molecules, although modulation of the PET process may contribute to the overall increases in emission observed.

We next examined a set of phosphorylated biomolecules structurally related to PAPS and PAP (Fig. 2). The molecules that contained a phosphate group (5′-AMP, 3′-AMP, CoA) together with PAPS and PAP all gave significant luminescence enhancements (at least 4-fold) at 613 nm. Structural analogues with a phosphate group bonded to an additional group but lacking an unconjugated phosphate (APS, dephospho-CoA) gave substantially lower enhancements in luminescence (*ca.* 2-fold). Similar trends in luminescence responses were obtained with the EupBOH2 and EuBn (Fig. S8†). Molecules with only one unconjugated phosphate group (PAPS, 5′-AMP, 3′-AMP, CoA) gave a smaller luminescence enhancement than PAP, which has two phosphates. We thus conclude that PAP coordinates the Eu(iii) complexes through both its phosphate groups. Conjugated phosphates (APS, dephospho-CoA) are also able to associate with the europium(iii) ion, but the resulting host–guest structures are significantly less emissive.

With a PAP-selective Eu(iii) complex in hand and a good understanding of its photophysical properties in aqueous solution, we proceeded to apply it to monitoring a sulfation reaction. We first evaluated the effect of our model acceptor substrate, heparin, on the luminescence of EumNH2 and found that this heavily sulfated polysaccharide produced no change in the emission of the complex either alone or in the presence of PAPS or PAP (Fig. S9†). This is consistent with our finding discussed above that sulfate does not interact with our Eu(iii) complex or affect its emission (Fig. S7†). The next step was to perform simulations of an enzyme reaction wherein our complexes were incubated with increasing molar ratios of PAP/PAPS, whilst keeping the total concentration of PAP and PAPS constant. In accord with the aim that our probes could ultimately be applied to high-throughput screening we conducted this work in 384-well plate format, taking advantage of the long emission lifetimes of the probes to record time-resolved measurements thereby increasing signal to noise ratio. All three Eu(iii) complexes gave linear increases in time-resolved emission intensity of the Δ*J* = 2 band (Fig. S10†) as the mole fraction of PAP increases. Furthermore, the extent of the emission increase (determined by the gradient) was consistent with the emission differences we observed in our earlier fluorimetry experiments (Fig. 1), confirming that our assay could transfer from quartz cuvette to polystyrene plate. These experiments also confirmed the aniline complex EumNH2 as our most responsive, and therefore preferred, probe.

**Fig. 3:**
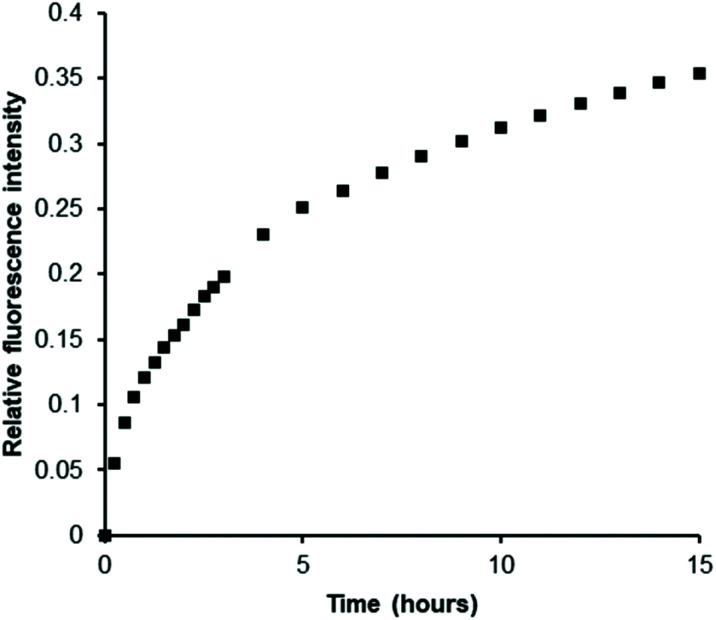
EumNH2 can be used to monitor sulfation of heparin by HS3ST1. Data is from a representative example of two independent experiments conducted in triplicate and corrected for emission decay with identical mixture except omitting heparin. Experiments used 5 μM probe, 250 μM PAPS, 500 μM heparin, 5 μM HS3ST1, in 50 mM TRIS at pH 7.4, rt.

Next, we prepared a heparan sulfotransferase HS3ST1 (tagged with glutathione-*S*-transferase (GST) for ease of purification, Fig. S11†) and incubated the recombinant enzyme with PAPS and an excess of porcine intestinal mucosal heparin, one of its natural substrates, in the presence of our probe. While heparin molecules have already been sulfated by this enzyme *in vivo* prior to its isolation we reasoned that, as with all HSST reactions, sulfation of acceptor sites (in this instance on the C3 hydroxyl of glucosamine residues) is only partial.^39^ Indeed, bovine intestinal heparin has been shown to be a substrate *in vitro* for this enzyme.^40^ We were delighted to observe a gradual increase in luminescence as PAPS was consumed and PAP was generated (Fig. 3). This demonstrates the ability of our Eu(iii)-based anion receptor to function in a biological sulfation assay and thus renders the first example of real-time monitoring of a heparan sulfotransferase that is in principle independent of both substrate and product. Confirmation of the conversion of PAPS to PAP during the enzyme reaction was given by ^1^H NMR spectroscopy (Fig. S12†).

## Conclusion

We have demonstrated proof-of-concept for the use of a new europium(iii)-based anion receptor to monitor in real-time the activity of a heparan sulfotransferase. Previous assays^24–27^ have focussed on the macromolecular products of these reactions and required labour-intensive techniques and specialist equipment (and often a radiolabel) or use a coupled assay. In contrast our method directly measures the small anionic reaction partner and product, is independent of the acceptor, is label-free and relies solely on a standard plate reader. Our supramolecular approach to enzymatic monitoring exploits the ability of complex EumNH2 to bind and discriminate between the structurally similar phosphoanions, PAP and PAPS, whilst showing no interference from sulfated biomolecules present in the bioassay.

Whilst this work brings the carbohydrate sulfotransferases, which are known to be druggable targets,^27^ one step closer to high throughput inhibitor screening, our assay has some shortcomings. As noted above, the ability of the aniline nitrogen lone pair in EumNH2 to act as a quencher of the europium(iii) excited state means that our most selective probe is only weakly emissive. Future work will further investigate the interaction of target anions with EumNH2, including a possible role for the amine group, and so attempt to design and synthesise molecules that combine the selectivity of EumNH2 with the higher inherent brightness of the boronic acid probe. These studies are underway in our laboratories and will be reported in due course.

## Experimental

### General considerations

All chemicals were purchased from Cayman, Fluorochem, SiChem or Sigma-Aldrich and used without further purification.

NMR spectroscopy was carried out in the stated deuterated solvent using a JEOL ECS-400 (^1^H at 399.78 MHz, ^13^C at 100.53 MHz) or a JEOL–ECS-500 spectrometer (^1^H at 500.16 MHz, ^13^C at 125.77 MHz) spectrometer at 293 K. Chemical shifts are expressed in ppm and are adjusted to the chemical shift of the residual NMR solvent resonances (CDCl_3_: ^1^H *δ* = 7.26 ppm, ^13^C *δ* = 77.16 ppm or CD_3_OD: ^1^H *δ* = 3.31 ppm, ^13^C *δ* = 49.00 ppm).

All complexes were stored as 1 mM solutions in distilled water at −20 °C and diluted solutions prepared from these.

UV-Vis spectroscopy was performed using a Shimadzu UV-1800 instrument. Molar extinction coefficients were calculated using the Beer–Lambert law.

Luminescence spectroscopy was performed using a Camlin Photonics luminescence spectrometer with FluoroSENS version 3.4.7.2024 software. Emission spectra were obtained using a 40 μL Hellma® Analytics quartz cuvette with excitation at 322 nm and reading emission in the range 550–720 nm using an integration time of 0.1s, increment of 1 nm and excitation and emission slits of 0.5 nm.

Quantum yields were measured using quinine sulfate in 0.05 M H_2_SO_4_ as a standard (*Φ*_em_ = 0.58, *λ*_ex_ = 350 nm).^41^ Plate-based assays were conducted in FisherBrand black, polystyrene 384-well plates with a maximum volume of 44 μL per well.

### Complex synthesis

Boronic acid and parent complexes were prepared as previously described.^29^ Complex EumNH2 was prepared as follows (for atom numbering see Fig. S13†):

#### Compound 2

To a stirred solution of phenol 1 (200 mg, 1.15 mmol) in DMF (10 mL) was added K_2_CO_3_ (235 mg, 1.7 mmol). After 5 minutes bromide (395 mg, 1.38 mmol) was added dropwise as a solution in DMF (2 mL). After 1 hour the reaction was diluted with water (50 mL) and extracted with EtOAc (3 × 15 mL). Combined organics were washed with brine, dried over MgSO_4_ and evaporated to a yellow oil that was purified by chromatography over SiO_2_ (20 g) eluting with 10–25% EtOAc/40–60 petroleum ether. Relevant fractions were combined, evaporated and dried under high vacuum to yield product 2 as an off-white solid (449 mg, 1.01 mmol, 88%). ^1^H NMR (500 MHz, CDCl_3_):1.49 (9H, s, H^18^), 5.44 (2H, s, H^9^), 6.55 (1H, bs, NH), 7.12 (1H, dd, *J* = 7.8, 1.1 Hz, H^7^), 7.20 (1H, m, H^15^), 7.27–7.32 (2H, m, H^6^, H^13^), 7.45 (1H, dd, *J* = 8.3, 1.0 Hz, H^5^), 7.52 (1H, m, H^14^), 7.60 (1H, s, H^11^), 8.06 (1H, d, *J* = 8.4 Hz, H^3^), 8.26 (1H, d, *J* = 8 Hz, H^4^), 10.31 (1H, d, *J* = 0.9 Hz, H^19^). ^13^C NMR (126 MHz, CDCl_3_): 28.4 (C^18^), 71.0 (C^9^), 80.7 (C^17^), 111.1 (C^7^), 116.8 (C^11^), 117.9 (C^3^), 118.0 (C^13^), 120.0 (C^5^), 121.5 (C^15^), 129.5 (C^14^), 129.7 (C^6^), 131.5 (C^4a^), 137.3 (C^4^), 137.7 (C^10^), 138.9 (C^12^), 140.4 (C^8a^), 151.7 (C^8^), 152.8 (C^2^), 155.2 (C^16^), 171.2 (C^19^). HRMS: C_22_H_23_N_2_O_4_^+^ requires 379.1652, found 379.1652.

#### Compound 3

To a stirred suspension of aldehyde 2 (430 mg, 1.14 mmol) in MeOH (20 mL) under 1 atmosphere of N_2_ and cooled in an ice-bath was added NaBH_4_ (64 mg, 1.5 mmol) causing effervescence. After 1 hour the reaction was diluted with sat. aq. NH_4_Cl (20 mL) and extracted with EtOAc (3 × 15 mL). Combined organics were washed with brine, dried over MgSO_4_ and evaporated to a yellow oil that was purified by chromatography over SiO_2_ eluting with EtOAc. Relevant fractions were combined, evaporated and dried under high vacuum to yield product 3 as an off-white solid (244 mg, 0.64 mmol, 56%). ^1^H NMR (500 MHz, CDCl_3_): 1.49 (9H, s, H^18^), 4.55 (1H, bs, OH), 4.92 (2H, s, H^19^), 5.31 (2H, s, H^9^), 6.65 (1H, bs, NH), 7.08 (1H, dd, *J* = 5.4, 3.6 Hz, H^7^), 7.18 (1H, d, *J* = 8.4 Hz, H^15^), 7.28 (1H, m, H^14^), 7.30–7.34 (2H, m, H^3^, H^13^), 7.36–7.39 (2H, m, H^5^, H^6^), 7.56 (1H, bs, H^11^), 8.08 (1H, d, *J* = 8.4 Hz, H^4^). ^13^C NMR (126 MHz, CDCl_3_): 28.4 (C^18^), 64.6 (C^19^), 70.8 (C^9^), 80.6 (C^17^), 111.1 (C^7^), 117.0 (C^11^), 117.9 (C^13^), 119.0 (C^3^), 120.1 (C^5^), 121.5 (C^15^), 126.5 (C^14^), 128.9 (C^4a^), 129.3 (C^6^), 136.8 (C^4^), 138.1 (C^10^), 138.8 (C^12^) 139.1 (C^8a^), 152.8 (C^8^), 154.1 (C^2^), 158.2 (C^16^). HRMS: C_22_H_25_N_2_O_4_^+^ requires 381.1089, found 381.1807.

#### Compound 4

To a stirred solution of alcohol 3 (294 mg, 0.77 mmol) in DCM (10 mL) cooled in an ice-bath was added Et_3_N (162 μL, 1.16 mmol) followed by MsCl (72 μL, 0.93 mmol). After 1 hour fresh portions of Et_3_N (81 μL, 0.58 mmol) and MsCl (36 μL, 0.46 mmol) were added. After a further 1 hour the reaction was washed with water (2 × 15 mL), then with brine (20 mL), dried over MgSO_4_ and evaporated to give a yellow gum which was used without further purification. ^1^H NMR (500 MHz, CDCl_3_):1.49 (9H, s, H^18^), 3.14 (3H, s, H^20^), 5.33 (2H, s, H^19^), 5.57 (2H, s, H^9^), 6.61 (1H, bs, NH), 7.08 (1H, dd, *J* = 7.0, 1.9 Hz, H^7^), 7.15 (1H, d, *J* = 7.3 Hz, H^15^), 7.26–7.35 (2H, m, H^13^, H^14^), 7.38–7.45 (2H, m, H^5^, H^6^), 7.55 (1H, d, *J* = 8.5 Hz, H^3^), 7.56 (1H, bs, H^11^), 8.18 (1H, d, *J* = 8.5 Hz, H^4^).

To a stirred solution of mesylate (445 mg, 0.95 mmol) in MeCN (40 mL) was added K_2_CO_3_ (394 mg, 2.85 mmol) followed by DO2A-*tert* butyl ester (380 mg, 0.95 mmol) and the mixture stirred at 60 °C for 18 hours. The reaction mixture was cooled and centrifuged. The liquours were decanted and the solid washed with DCM (2 × 10 mL). Combined liquids were evaporated to give a yellow gum which was purified by column chromatography over SiO_2_ (40 g) eluting with EtOAc then with 95 : 5 : 0.5 to 90 : 10 : 1 to 80 : 20 : 5 DCM/MeOH/880 NH_3_. Relevant fractions were combined and evaporated to yield product 4 as an off-white foam (513 mg, 0.67 mmol, 71%). ^1^H NMR (500 MHz, CD_3_OD): Compound 4 present as two rotamers in approximate ratio 4 : 1 1.37 and 1.48 (together 9H, s, H^18^, H^27^), 2.38–3.10 (20H, m, H^20–24^), 3.60 and 3.93 (together 2H, s, H^19^), 5.18 and 5.31 (together 2H, s, H^9^), 6.70–7.89 (9H, m, NH, H^3^, H^4–7^, H11, H^13–15^), 8.06 and 8.31 (together 1H, d, *J* = 8.5 Hz, H^4^). ^13^C NMR (126 MHz, CD_3_OD): some peaks doubled due to presence of rotamers 31.0 and 31.3 (C^18^ and C^27^), 47.8, 52.8, 53.4 and 57.4 (C^20–23^), 59.0 and 59.1 (C^24^), 63.8 and 64.6 (C^19^), 75.2 and 75.6 (C^9^), 83.7 and 85.1 (C^26^), 85.0 and 85.3 (C^17^) 114.3 and 115.2 (C^7^), 123.2 (C^11^), 123.5 (C^13^), 124.3 and 124.5 (C^3^), 127.5 and 127.6 (C^15^), 130.3 and 130.7 (C^14^), 132.3 and 132.6 (C4^a^), 132.8 and 133.4 (C^6^), 140.6 and 141.1 (C^4^), 141.4 (C^10^), 143.3 and 143.5 (C^12^), 144.3 (C^8^), 157.7 (two peaks, C^2^), 157.9 (C^8a^) 161.4 and 161.5 (C^16^), 174.7 and 174.9 (C^25^). HRMS: C_42_H_62_N_6_O_7_^+^ requires 763.4674, found 763.4753

#### Compound 5

To a stirred solution of protected ligand 4 (500 mg, 0.66 mmol) in DCM (7.5 mL) was added TFA (2.5 mL) and stirring continued for 18 hours. After this time the solvent was removed *in vacuo* and the residue co-evaporated from DCM (5 × 10 mL) to yield a brown gum corresponding to ligand 5 as the 3TFA salt (589 mg, 0.66 mmol, quant.), which was used in the next step without further purification. ^1^H NMR (400 MHz, CD_3_OD): 2.71–4.25 (20H, m, H^20–24^), 4.77 (2H, s, H^19^), 5.37 (2H, s, H^9^), 7.20 (1H, H^15^), 7.26 (1H, dd, *J* = 5.7, 3.3 Hz, H^13^), 7.34–7.59 (H^5–7^, H^11^, H^14^), 7.62 (1H, d, *J* = 8.5 Hz, H^3^) 8.34 (1H, d, *J* = 8.5 Hz, H^4^). ^13^C NMR (126 MHz, CD_3_OD) 42.5 (C^24^), 48.8, 49.3, 52.1, 52.9 (C^20–23^) 57.4 (C^19^), 69.9 (C^9^), 110.4 (C^7^), 116.8 (q, *J* = 291 Hz, TFA CF_3_) 119.4 (C^13^), 119.7 (C^3^), 119.8 (C^11^), 122.3 (C^5^), 124.3 (C^15^), 127.6 (C^14^) 127.8 (C^6^), 129.2 (C^10^), 129.9 (C^4a^), 137.8 (C^4^), 139.35 (C^12^), 139.42 (C^8a^), 150.2 (C^8^), 154.1 (C^2^), 161.5 (q, *J* = 35 Hz, TFA C=O) 173.7 (C^25^) HRMS: C_29_H_38_N_6_O_5_^+^ requires 551.2976, found 551.297.

### EumNH2

Ligand 5.3TFA (53 mg, 0.059 mmol) was dissolved in water (2 mL) and the pH adjusted to 8.7 with 2 M NaOH(aq). EuCl_3_·6H_2_O (23 mg, 0.063 mmol) was added causing a sharp drop in pH which was readjusted to 8.7 using 2 M NaOH(aq). The reaction mixture was heated at 60 °C for 18 hours maintaining pH ≥ 7.2. The reaction was centrifuged (5000 rpm, 5 min) and the supernatant purified by preparative reverse-phase HPLC (Waters 1525 Binary HPLC pump controlled by the Waters Breeze 2 HPLC system software, XBridge C18 (5 μm OBD 19 × 100 mm) column at a flow rate of 17 mL min^−1^, gradient: 0–100% MeCN in 50 mM NH_4_HCO_3(aq)_ over 15 min, detection using a Waters 2489 UV/Visible detector operating at 254 nm, *t*_R_ = 6.07 min). Solvent was removed by evaporation and then lyophilisation to yield EumNH2 as a white solid (3.7 mg, 0.005 mmol, 9%). HRMS: C_29_H_36_^153^EuN_6_O_5_^+^ requires 701.1948, found 701.1951. *λ*_max_ = 322 nm, *ε* = 2500 M^−1^ cm^−1^, *φ* = 0.3%, *τ*_H_2_O_ = 0.05 ms, *τ*_D_2_O_ = 0.07 ms.

### Preparation of GST-HS3ST1

The cDNA fragment encoding the sulfotransferase HS3ST1 (O35310_48-311) used previously by others^42^ was purchased from Thermo Fisher Scientific (Thermo Fisher, UK). EcoRI and NotI were used as restriction sites for inserting the HS3ST1 encoding fragment into the pGEX4T3 vector. HS3ST1 was expressed in C41 (DE3) strain of *Escherichia coli* and induced with 200 μM isopropyl 1-thio-β-d-galactopyranoside (IPTG) at 22 °C overnight.37 The cells were collected by centrifugation for 15 min at 4150*g* and resuspended in lysis buffer (100 mM NaCl, 50 mM Tris-Cl, pH 7.4) prior to cell breakage by sonication (six cycles of 30 s sonication and 30 s rest, on ice). Purification was achieved by applying the lysate sequentially to two columns. First, a 2 mL glutathione resin (Genscript Biotech Corporation, Netherlands) self-packed column, washed with 100 mM NaCl, 50 mM Tris, pH 7.4 was eluted with 10 mM reduced glutathione, 100 mM NaCl, 50 mM Tris, pH 7.4. Second, the eluted fraction was applied to a 1 mL heparin (Affi-Gel hep, Bio-Rad, UK) self-packed column, washed with 50 mM NaCl, 50 mM Tris, pH 7.4 and, and eluted with 600 mM NaCl, 50 mM Tris, pH 7.4. Protein was quantified from its absorbances at 280 nm, using the extinction coefficient calculated for the amino acid sequence, snap frozen in liquid nitrogen and stored in aliquots at −80 °C.

### Luminescence experiments

Luminescence spectra were recorded on a Camlin Photonics luminescence spectrometer with FluoroSENS version 3.4.7.2024 software. Emission spectra were obtained using a 40 μL Hellma Analytics quartz cuvette. Excitation light was set at 322 nm and emission recorded in the range 550–720 nm using an integration time of 0.5 seconds, increment of 1.0 nm, excitation slit of 0.2 nm and emission slit of 0.5 nm.

Emission lifetime measurements were performed on the FluoroSENS instrument. Measurements were taken of 40 μL of 5 μM samples of Eu(iii) complexes in 50 mM TRIS at pH 7.4. Measurements were obtained by indirect excitation of the Eu(iii) ion *via* the quinoline antennae using a short pulse of light at 322 nm followed by monitoring the integrated intensity of the light emitted at 620 nm, with 500 data points collected over a 5 millisecond time period. The decay curves were plotted in Origin Labs 2019 version 9.6.0.172, and fitted to the equation:

1 *I* = *A*_0_ + *A*_1_e^−*kt*^

where *I* is the intensity at time, *t*, following excitation, *A*_0_ is the intensity when decay has ceased, *A*_1_ is the pre-exponential factor and *k* is the rate constant for the depopulation of the excited state.

The hydration state, *q*, of the Eu(iii) complexes was determined using the modified Horrocks equation:^35^

2 *q*(Eu) = 1.2 (1/*τ*_H_2_O_ − 1/*τ*_D_2_O_ − 0.25 − 0.075*n*)

where *τ*_H_2_O_ and *τ*_D_2_O_ are the emission lifetime times in water and D_2_O, respectively, and *n* is the number of carbonyl-bound amide NH groups.

### Anion binding studies

Stock solutions of Eu(iii) complexes were diluted to 5 μM using 50 mM TRIS buffer. 40 μL was placed in a quartz cuvette and the emission spectrum recorded. 1 μL of 10 mM anion solution in distilled water was added (final anion concentration 250 μM) and the emission recorded again. For inorganic anions (Fig. S5†) 1.6 μL of a 25 mM solution in HEPES was used, final concentration 1 mM.

### Anion binding titrations

Anion binding titrations were carried out in 50 mM TRIS buffer at pH 7.4. Stock solutions of PAPS and PAP containing Eu(iii) complex (5 μM) were made up at 0.1, 1 and 5 mM anion. The appropriate anion stock solution was added incrementally to 40 μL of Eu(iii) complex (5 μM) and the emission spectrum was recorded after each addition. The ratio of emission bands 605–630 nm/585–600 nm (Δ*J* = 2/Δ*J* = 1) was plotted as a function of anion concentration. The data was analysed using a nonlinear least-squares curve fitting procedure, based on a 1 : 1 binding model described by the equation:
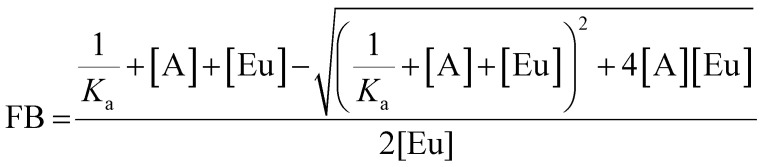
where FB is the fraction bound, calculated by (*I* − *I*_0_)/(*I*_1_ − *I*_0_) where *I* is the emission intensity at [A], *I*_0_ is the initial emission intensity, and *I*_1_ is the final emission intensity. [A] is the total concentration of anion in solution, [Eu] is the total concentration of Eu(iii) complex, *K*_a_ is the apparent binding constant.

### Microplate-based sulfotransferase simulations

Varying ratios of a solution of PAPS and PAP containing a 5 μM Eu(iii) complex in 50 mM TRIS at pH 7.4 were added to a 384-well plate, in triplicate, to a total well volume of 40 μL. The plate was incubated for 10 minutes prior to reading. Time-resolved emission intensities were recorded in the range 610–630 nm (integration time of 60–400 μs) with excitation at 292–366 nm. The mean of the triplicate intensity values was plotted against the percentage of PAP. Error bars indicate the standard error in the mean value.

### Microplate-based sulfotransferase reactions

1.5 μL of 200 μM EumNH2 was added to 58.5 μL of 20 μM GST-HS3ST1 and the mixture incubated at rt for 5 minutes. 6 × 30 μL of a 5 μM solution of EumNH2 in 50 mM TRIS pH 7.4 were placed in a 384-well plate and to these were added the mixture of EumNH2 and GST-HS3ST1 (final concentrations both 5 μM). 3 μL of either 50 mM TRIS pH 7.4 or 100 mg mL^−1^ heparin were added to 3 wells each and the plate allowed to incubate at rt for 5 minutes. 1 μL of PAPS (10 mM in distilled water) was added to each well (final concentration 250 μM). The wells were scanned every 15 minutes for 3 hours then every hour for an additional 20 hours exciting at 292–364 nm and measuring emission at 620 ± 5 nm. Emission change was calculated by normalising to the emission at *t* = 0, averaging over replicates and subtracting the signal from wells with TRIS from the signal from wells with heparin.

## Conflicts of interest

There are no conflicts to declare.

## Supplementary Material

OB-020-D1OB02071D-s001
