# Supplementary material for: Anion binding to a cationic europium(iii) probe enables the first real-time assay of heparan sulfotransferase activity
Source: Org Biomol Chem. 2021 Dec 8;20(3):596–605. doi: 10.1039/d1ob02071d (PMC8767414; doi:10.1039/d1ob02071d)
Supplement: OB-020-D1OB02071D-s001 [file OB-020-D1OB02071D-s001.pdf]

Electronic Supplementary Material (ESI) for Organic & Biomolecular Chemistry.
This journal is © The Royal Society of Chemistry 2021

Supporting Information

Anion binding to a cationic europium(III) probe enables the first real-time assay of heparan sulfotransferase activity

Simon Wheeler, Colum Breen, Yong Li, Sarah H. Hewitt, Erin Robertson, Edwin A. Yates, Igor L. Barsukov, David G. Fernig and Stephen J. Butler*

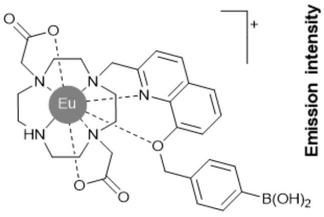

EupBOH2

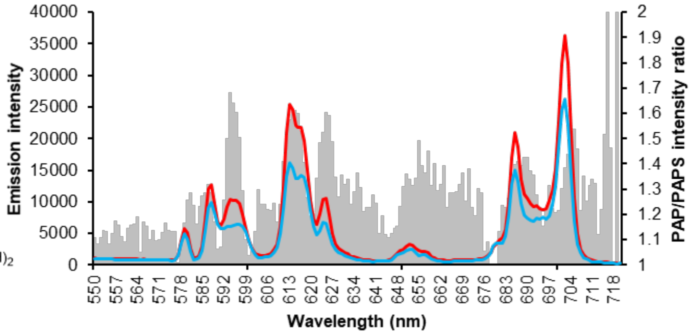

PAP/PAPS emission ratio at 613 nm	1.6
PAP/PAPS total emission ratio	1.3
PAP/PAPS (ΔJ=2)/(ΔJ=1) emission ratio	1.1

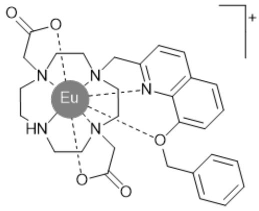

EuBn

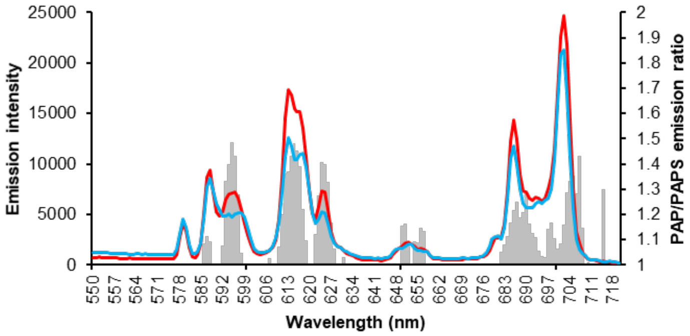

PAP/PAPS emission ratio at 613 nm	1.4
PAP/PAPS total emission ratio	1.1
PAP/PAPS (ΔJ=2)/(ΔJ=1) emission ratio	1.2

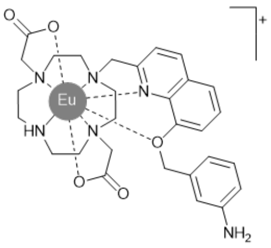

EumNH2

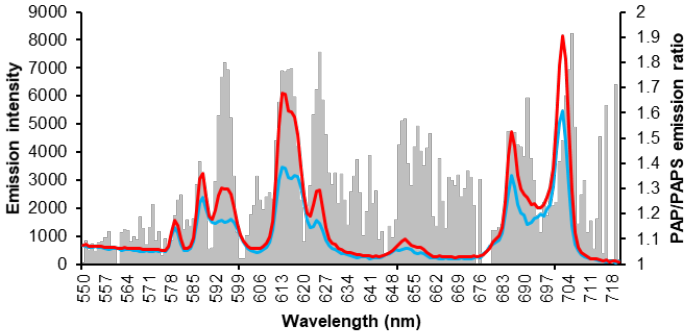

PAP/PAPS emission ratio at 613 nm	1.8
PAP/PAPS total emission ratio	1.4
PAP/PAPS (ΔJ=2)/(ΔJ=1) emission ratio	1.2

Figure S1. Selection of a luminescence method to assess the probes' discrimination. Plots show the emission spectra with PAP (red) and PAPS (blue) superimposed on a plot of the PAP/PAPS emission ratio for each wavelength (grey) demonstrating that, for each complex, emission at 613 nm offers the best combination of discrimination and intensity (and therefore sensitivity). The tables show that in every case the PAP/PAPS emission ratio at 613 nm was superior to the ratio of total emission and to the ratio of the (ΔJ=2)/(ΔJ=1) emission. ΔJ=1 band defined as 585-605 nm, ΔJ=2 band defined as 610-627 nm.

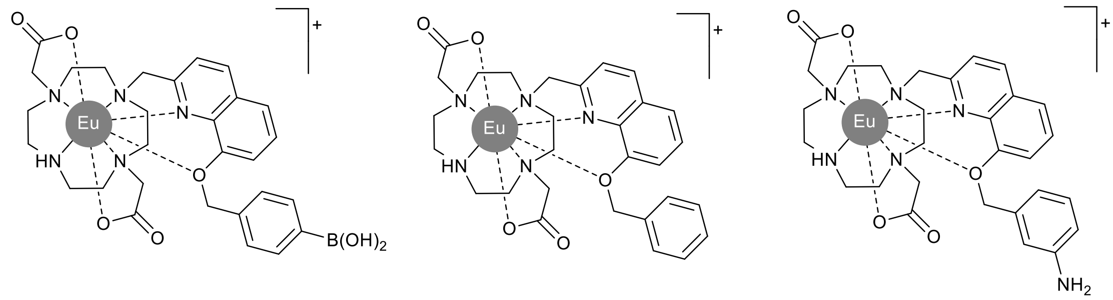

EupBOH2 EuBn EumNH2

A

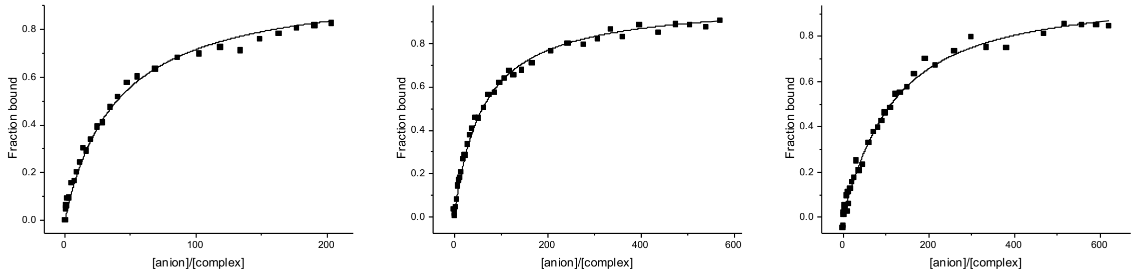

$\log K_A = 3.6 \pm 0.1$ $\log K_A = 3.7 \pm 0.3$ $\log K_A = 3.5 \pm 0.1$

B

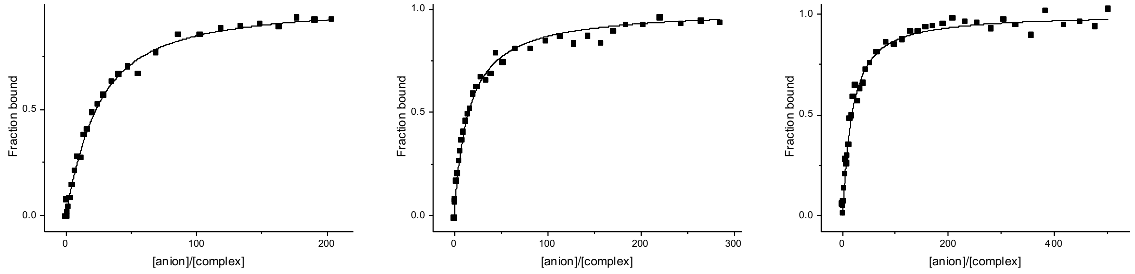

$\log K_A = 4.0 \pm 0.1$ $\log K_A = 4.0 \pm 0.2$ $\log K_A = 4.0 \pm 0.2$

Figure S2. Binding titrations with PAPS (**A**) and PAP (**B**) conducted using 5 μM probe, 50 mM TRIS, pH 7.4, 295 K. Plots are representative examples of two independent experiments; $\log K_a$ values are averages ± standard deviation.

		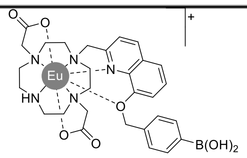	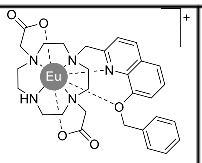	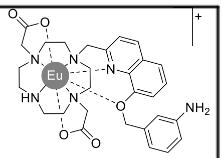
-	τ_{H2O}	0.17	0.20	0.05
	τ_{D2O}	0.21	0.24	0.06
	q	1.29	0.76	-[a]
PAPS	τ_{H2O}	0.32	0.33	0.14
	τ_{D2O}	0.42	0.41	0.17
	q	0.55	0.43	-[a]
PAP	τ_{H2O}	0.33	0.34	0.16
	τ_{D2O}	0.41	0.44	0.24
	q	0.40	0.41	-[a]
APS	τ_{H2O}	0.26	0.26	0.16
	τ_{D2O}	0.34	0.36	0.20
	q	0.73	0.94	-[a]

Table S1. Full lifetime data (expressed in ms) for complexes with added anions. Experiments were conducted in 50 mM TRIS using 50 μM complex and 1 mM anion; λ_{ex} = 322 nm, λ_{em} = 620 nm. [a] very short lifetimes prevented accurate estimation of *q* value.

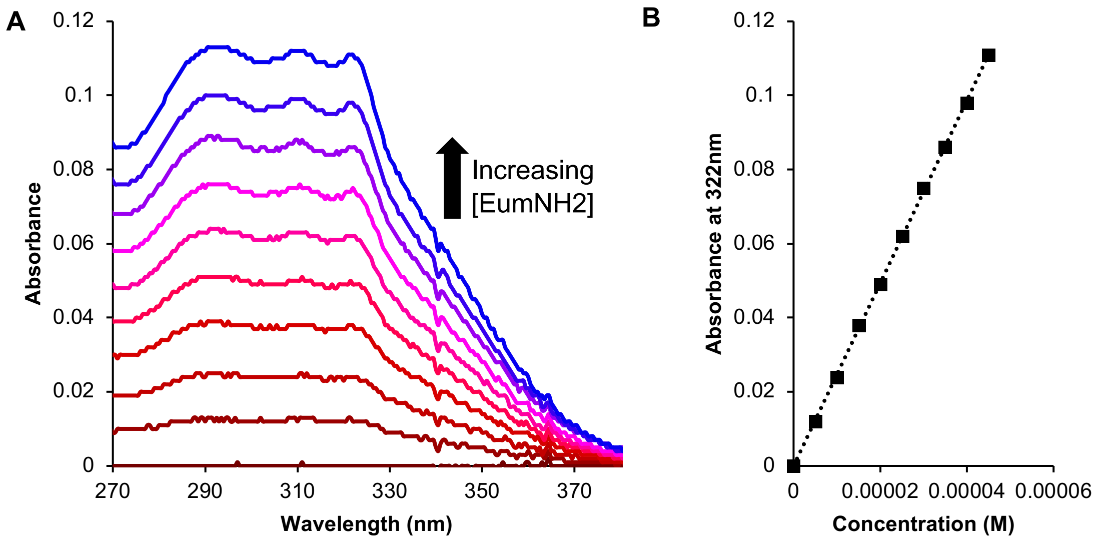

Figure S3. Absorbance data for complex EumNH2. (**A**) Absorption spectra at increasing concentration of complex. **B**) Absorbance maximum (322 nm) plotted against concentration. Experiments conducted in water at 295 K.

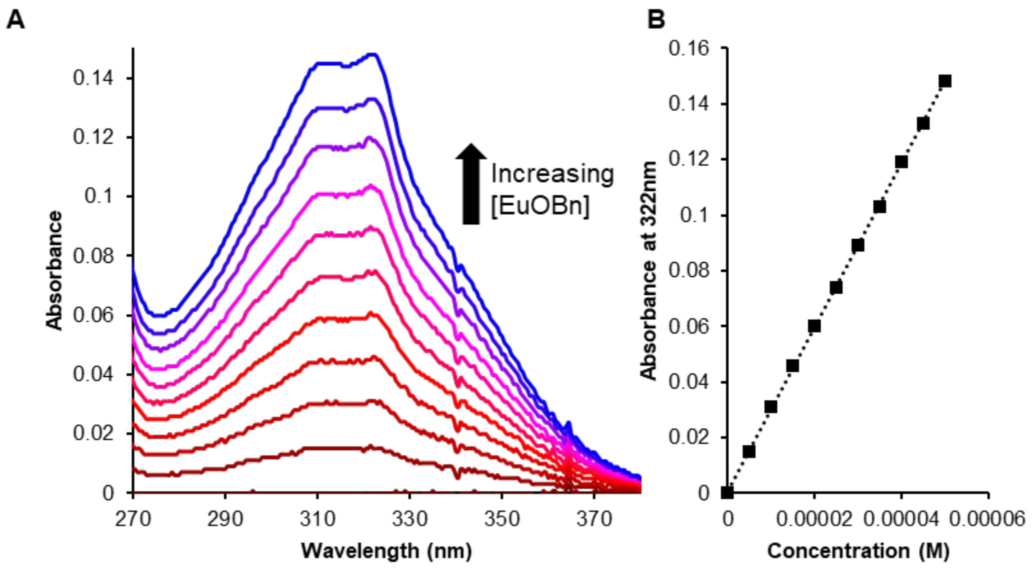

Figure S4. Absorbance data for complex EuBn. (**A**) Absorption spectra at increasing concentration of complex. (**B**) Absorbance maximum (322 nm) plotted against concentration. Experiments conducted in water at 295 K.

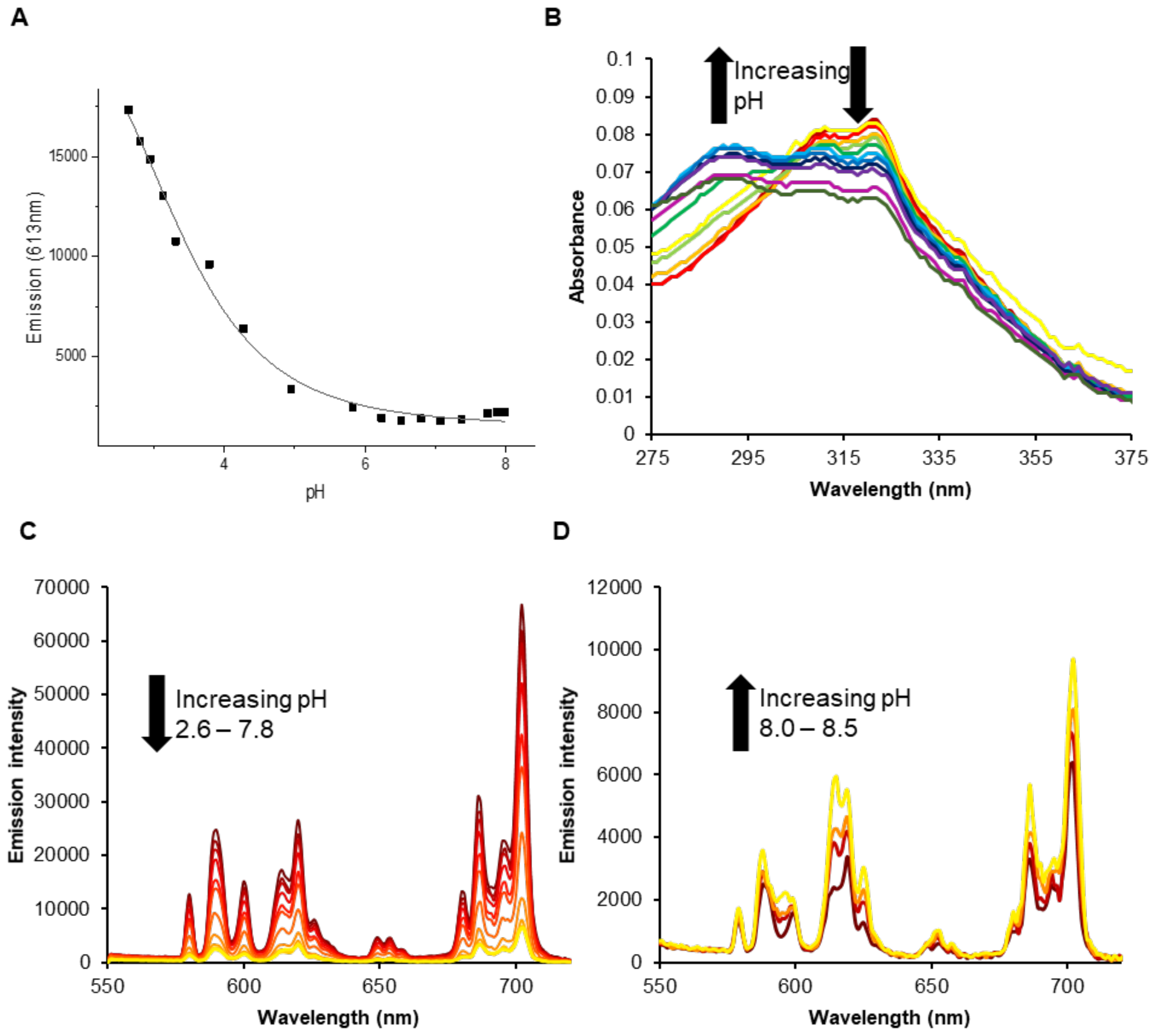

Figure S5. The effects of pH on the absorption and emission spectra of EumNH2. (A) Emission intensity at 613 nm of **EumNH2** is strongly pH-dependent but constant in the relevant range 6 – 7.8. (**B**) λ_{max} (322 nm) remains unchanged across the pH range and a high energy band centred at 292 nm emerges at higher pH. (**C**) Spectral form is unchanged in the pH range 2.6 – 7.8 (**D**) Above pH 7.8 spectral form changes are observed suggesting co-ordination of hydroxide. Experiment conducted using 5 µM probe in 50 mM TRIS at 295 K.

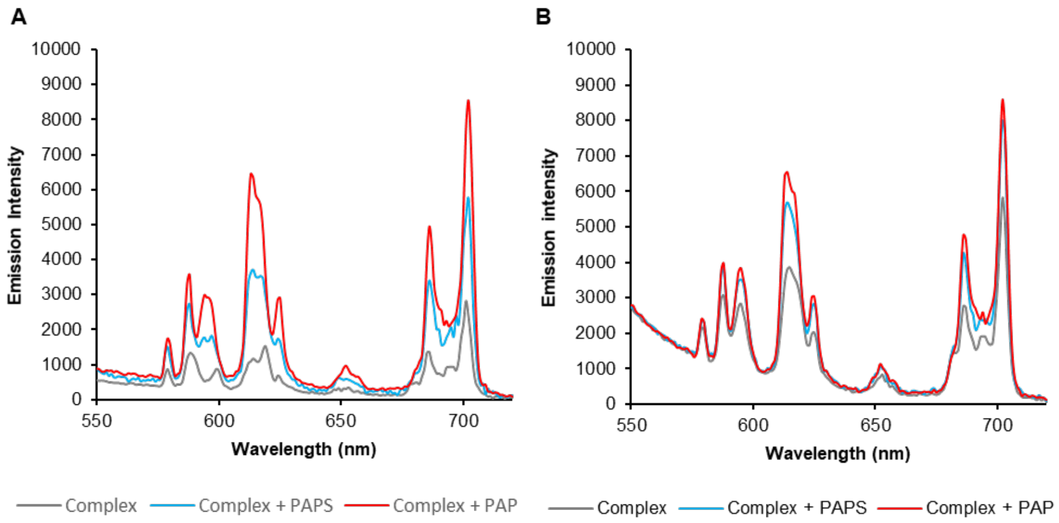

Figure S6. Emission of **EumNH2** in H_2O (**A**) and D_2O (**B**). Emission from the unbound complex (grey) is 2.9 times higher in D_2O but enhanced less by the addition of anions consistent with water displacement, rather than blocking PET, being the main pathway of emission enhancement. Experiments conducted using 5 µM probe, 250 µM in 50 mM TRIS, pH 7.4/pD 7.8, 293 K

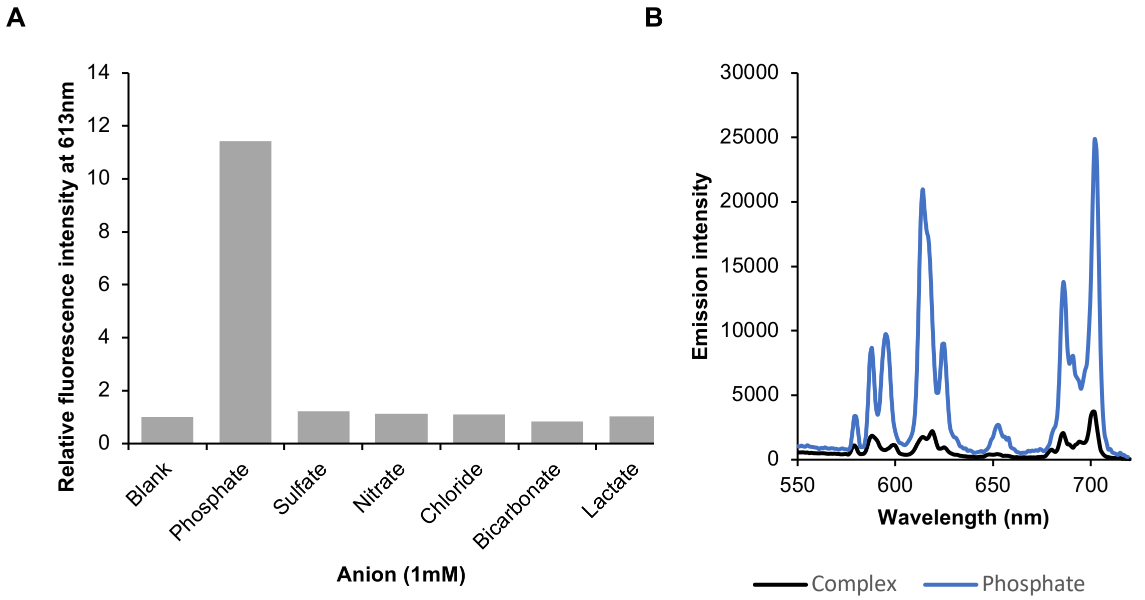

Figure S7. (**A**) Phosphate, but not other selected inorganic ions, gives significant enhancement in emission intensity of **EumNH2**. (**B**) Adding phosphate to **EumNH2** gives similar spectral form changes to those observed with PAPS and PAP. Experiments conducted using 5 μM probe, 1 mM anion in 50 mM TRIS at pH 7.4 and 295 K.

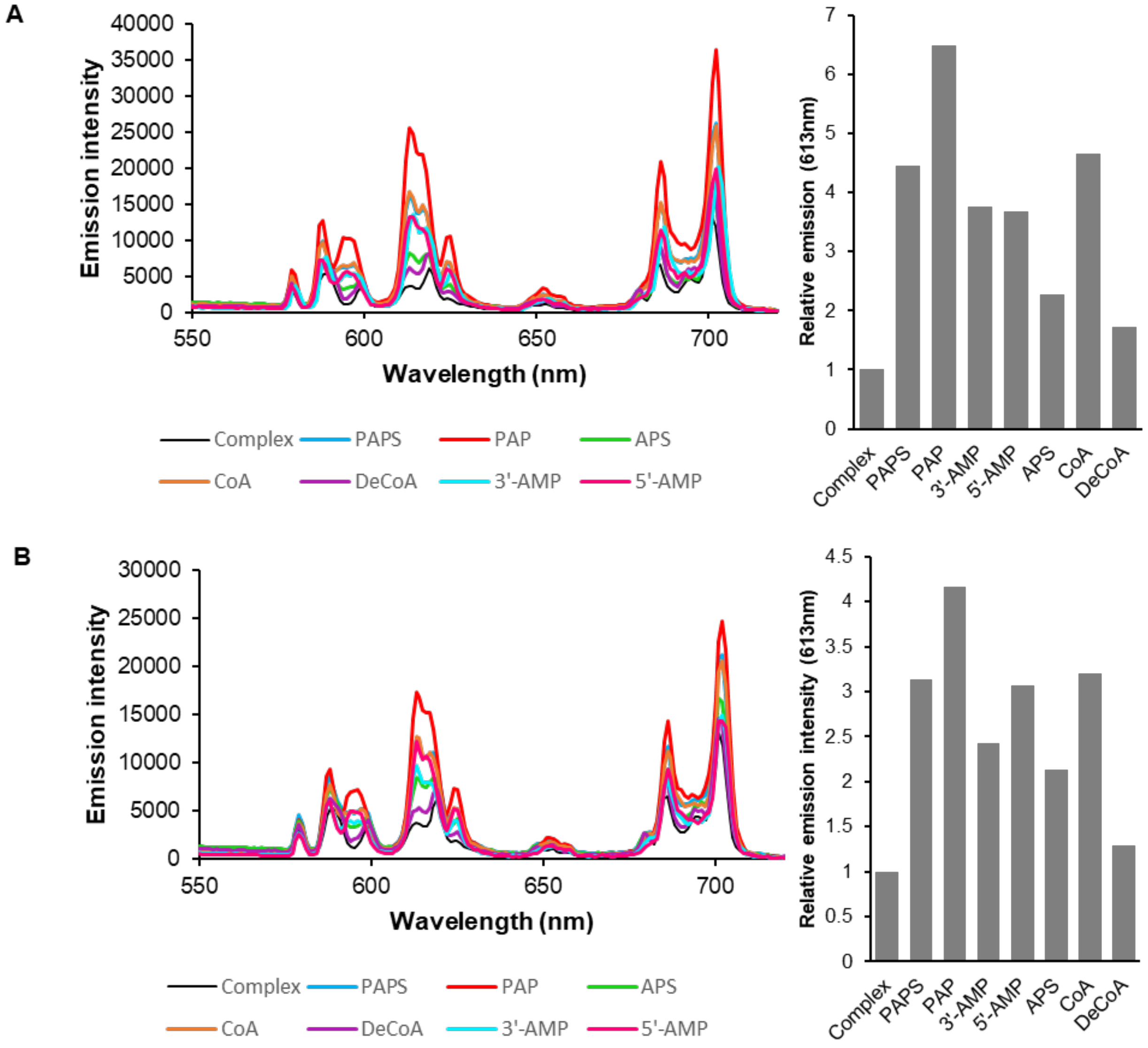

Figure S8. Phosphate, rather than phosphosulfate or hydroxyl groups are required for significant emission enhancement from **EupBOH2** (**A**) or **EuBn** (**B**). Experiments conducted using 5 μM probe, 250 μM anion in 50 mM TRIS at pH 7.4 and 295 K.

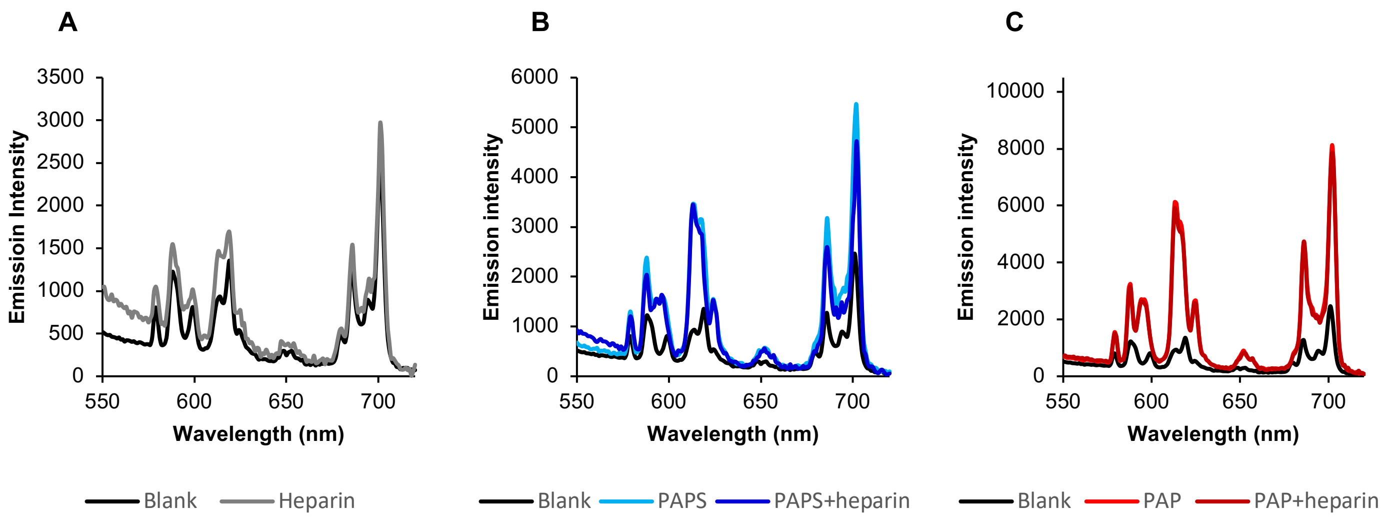

Figure S9. Effects of heparin on emission from **EumNH2**. The addition of heparin does not significantly affect the emission from **EumNH2** either in the absence of anion (**A**) or the presence of either anion of interest (**B**, **C**). Experiments conducted using 5 μM probe, 250 μM anion, 500 μM heparin in 50 mM TRIS, pH 7.4, 293 K.

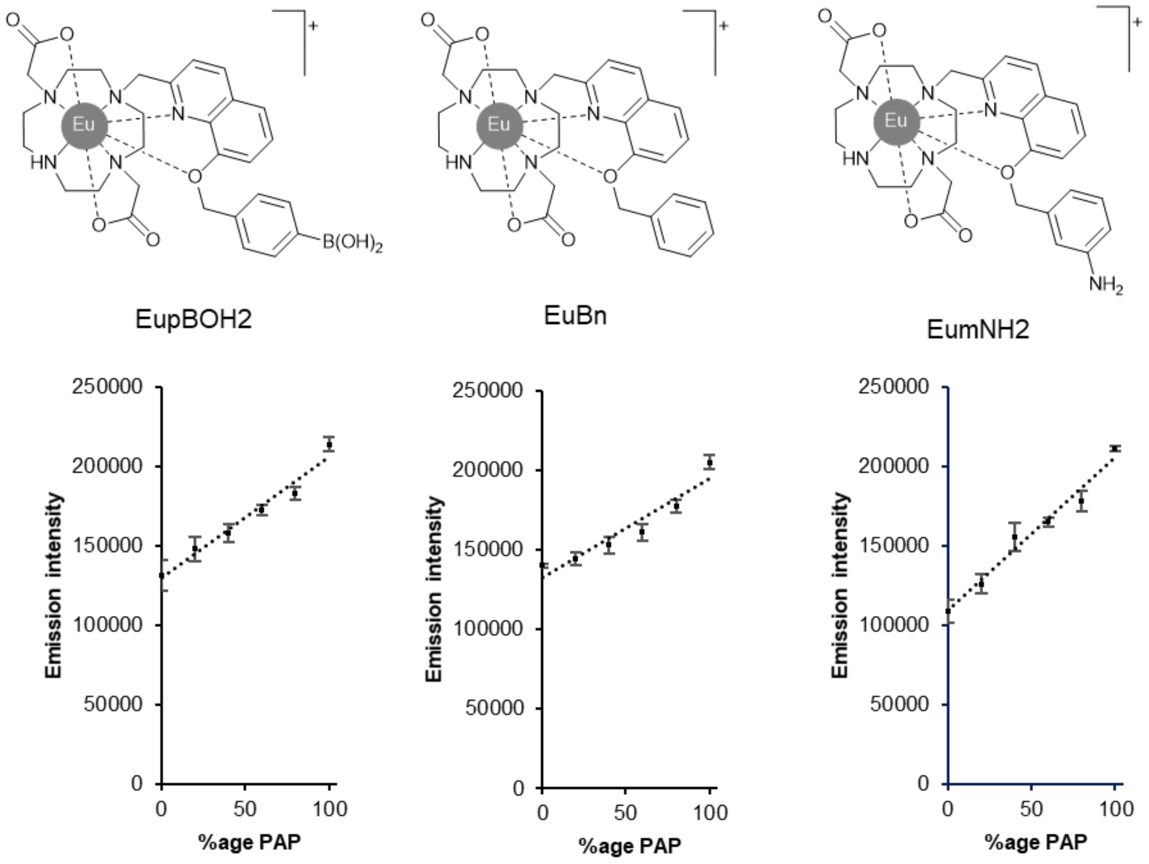

Figure S10. Enzyme simulation experiments confirm the ability of the probes to signal the PAPS/PAP ratio. Emission of the probes with mixtures of PAPS and PAP in varying proportions show linear correlations (r^2 = 0.965, 0.907 and 0.974 respectively). The gradients (1.6, 1.4 and 1.9, respectively) reflect the discriminatory power of each probe, which are consistent with emission experiments (Figure **1** of main article). All experiments conducted at 5 μM probe, total [anion] 250 μM, 5 mM $MgCl_2$ in 50 mM TRIS at pH 7.4 and 295 K. Data points are mean ± SEM of at least three independent experiments conducted in triplicate.

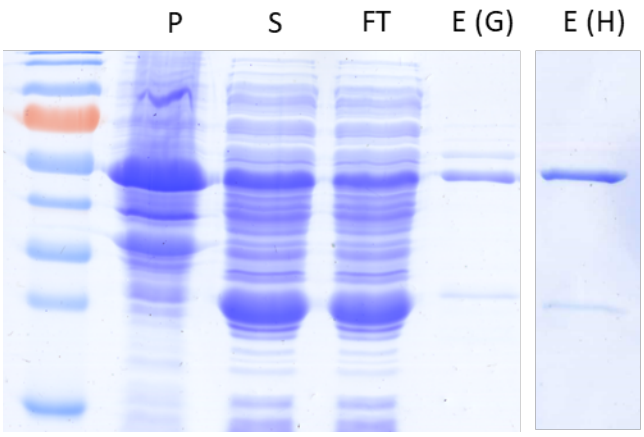

Figure S11. Expression and purification of GST-HS3ST1. The supernatant after cell breakage by sonication was applied to a glutathione resin column and the eluted fraction was applied to a heparin-affinity column. Samples were analysed by SDS-PAGE and Coomassie blue staining. P, bacterial pellet; S, supernatant following cell breakage; FT, unbound flow through fraction from the glutathione affinity column; E(G) eluate from the glutathione affinity column; E(H) eluate from the heparin affinity column.

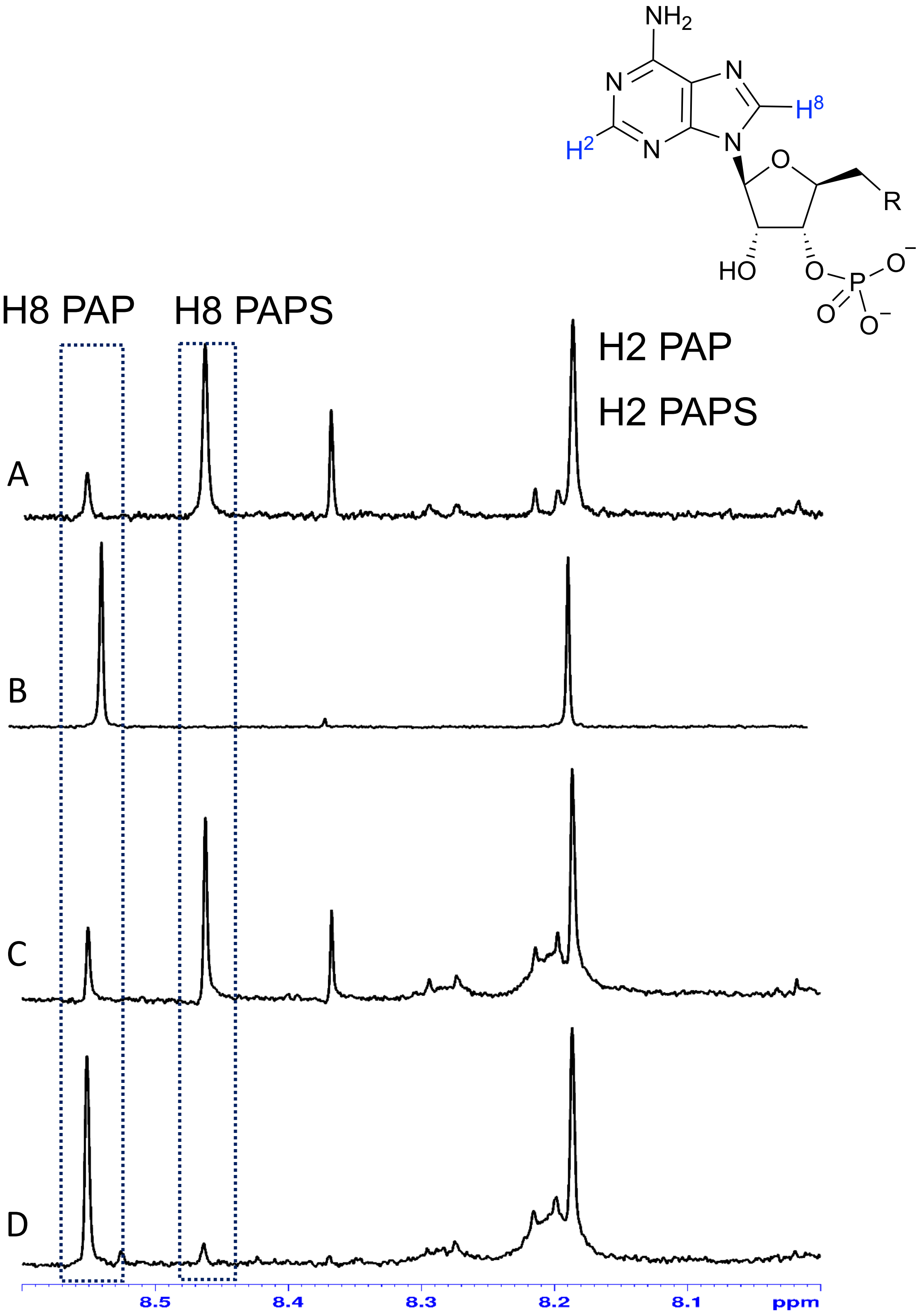

Figure S12. Partial ^{1}H NMR spectra showing conversion of PAPS to PAP by GST-HS3ST1. All samples were in 50 mM phosphate buffer and 170 mM NaCl, pH 7.4. A) the spectrum of 100 μM PAPS (8.462 ppm) and 1 μM GST-HS3ST1; B) the spectrum of 100 μM PAP (8.529 ppm); C) the spectrum of 100 μM PAPS, 2 mg/mL heparin and 1 μM GST-HS3ST1 after mixing; D) the spectrum of 100 μM PAPS, 2 mg/mL heparin and 1 μM GST-HS3ST1 after overnight incubation.

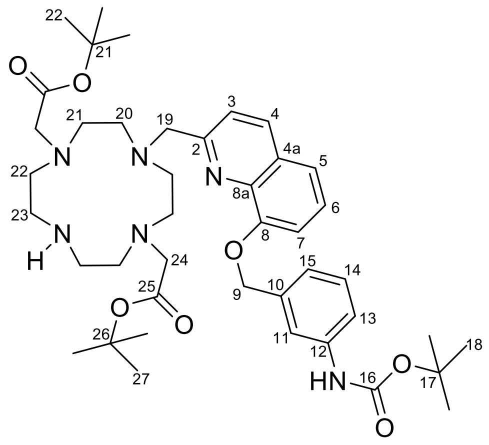

Figure S13. Atom numbering scheme for new compounds
